# Varroa Volatiles Offer Chemical Cues to Honey Bees for Initial Parasitic Recognition

**DOI:** 10.3390/biom15010066

**Published:** 2025-01-06

**Authors:** Qinglong Zhao, Xinning Wang, Ahsan Mustafa, Ying Wang, Hongfang Wang, Xuepeng Chi, Baohua Xu, Zhenguo Liu

**Affiliations:** Key Laboratory of Efficient Utilization of Non-Grain Feed Resources (Co-Construction by Ministry and Province), Ministry of Agriculture and Rural Affairs, Shandong Provincial Key Laboratory of Animal Nutrition and Efficient Feeding, College of Animal Science and Technology, Shandong Agricultural University, Tai’an 271018, China

**Keywords:** *Varroa destructor*, chemical ecology, volatile organic compounds, gas chromatography–mass spectrometry, electroantennography, headspace solid-phase microextraction

## Abstract

Olfaction mediated by the antennae is a vital sensory modality for arthropods and could be applied as a tool in pest control. The ectoparasitic mite *Varroa destructor* poses a significant threat to the health of the honey bee *Apis mellifera* worldwide and has garnered global attention. To better understand the chemical ecology of this host–parasite relationship, we collected and characterized the volatile organic compounds (VOCs) from *V. destructor* and used electroantennography (EAG) to record the responses of honey bee (*A. c. cerana* and *A. m. ligustica*) antennae to the different VOCs. Fifteen VOCs were detected from *V. destructor* using gas chromatography–mass spectrometry (GC-MS), which mainly contained ethyl palmitate, followed by isoamyl alcohol, nonanal, and ethyl oleate. The EAGs for ethyl palmitate were higher at the lowest stimulus loading (5 μg/μL in liquid paraffin) in *A. c. cerana* compared to *A. m. ligustica*, suggesting that *A. c. cerana* may have acute sensitivity to low concentrations of some VOCs from *V. destructor*. After exposure to ethyl palmitate for 1 h, the relative expression levels of *AcerCSP1* and *AcerOBP21* in *A. c. cerana* significantly increased, as well as the level of *AmelCSP1* in *A. m. ligustica*, while *AmelOBP8* showed no significant changes. The results indicate that the EAG response was influenced by the VOC composition and concentration. *A. c. cerana* tended to be more responsive than *A. m. ligustica* to the VOCs of *V. destructor*. Our findings offer a deeper understanding of how bees recognize *V. destructor*, potentially using ethyl palmitate as a chemical cue.

## 1. Introduction

Managed honey bees play a central role in crop yield and food security as pollinators; consequently, bees contribute significantly to global food production with an estimated economic value of USD 235 to USD 577 billion annually [1,2]. Moreover, approximately one-third of total human dietary supply relies on bee-pollinated crops, emphasizing the critical role of bees in our food system [3]. However, various beekeepers have recently faced high losses in managed honey bee colonies [4]. Recently, concerns over the plight of honey bees have become heightened due to the pollinator shortage.

To date, the exact reasons for bee colony losses remain unknown. Colony losses are partly attributed to diverse biotic and abiotic stresses, including parasitic *V. destructor* mite infestations, insecticides, pathogenic disease outbreaks, and a wide range of other possible factors [5]. As well as the continued decline in natural habitats, *V. destructor* has posed a serious threat to honey bee health and welfare since it was introduced to the Western honey bee *A. mellifera* [6]. Currently, *V. destructor* is the most important ectoparasite of *A. mellifera* [7]. This serious pest inflicts more damage and economic costs than all other apicultural diseases [8]. Varroa is responsible for the rapid decline in colony health and eventually colony death if left untreated [9,10]. Although various attempts were made to mitigate recent losses, there are currently no safe and efficient prevention measures available. Unfortunately, beekeepers worldwide still struggle to control varroa mites. Despite the ongoing risks of miticide resistance and the potential impact on bee health [11], the control of *V. destructor* in commercial hives continues to rely heavily on chemicals [12].

This stands in contrast with the Eastern honey bee *Apis cerana* (*A. cerana*), the original host of *V. destructor*, which exhibits robust mite resilience owing to its ability to detect and eliminate mites in adult bees through grooming behavior [13]. Jung et al. found that *A. cerana* has more olfactory sensilla in the antennae than *A. mellifera*, allowing it to respond more sensitively to various volatile compounds [14].

Chemical communication is involved in a wide range of physiological processes, including interactions between hosts and parasites [15]. Moreover, chemical communication is the most common approach employed by honey bees across different castes and individuals within the colony [16], such as the well-known brood ester pheromone [17] and (E)-*β*-ocimene [18] emitted by a live brood or oleic acid emitted by dead individuals [19]. Nazzi et al. reported that chemical communication is by far the most important channel that the mite uses to adapt its activity to the environment and the host [7]. *V. destructor* relies on its olfaction to detect semiochemicals directly emitted by the host or coming from the colony matrices [20,21]. However, the volatile composition of *V. destructor* remains poorly defined. Furthermore, the responses of honey bees to the mixed and individual volatile compounds of *V. destructor* have not yet been thoroughly investigated.

As far as we know, the electrophysiologically active volatile organic compounds (VOCs) from *V. destructor* have not been well explored. We suspect that the VOC collections may contain odors as important chemical cues in parasitic recognition. Previous studies have demonstrated that higher resistance to *V. destructor* could be related to better olfactory sensing in the host [22]. There is accumulating evidence indicating that the antennae of worker bees may play a key role in varroa-sensitive hygiene (VSH) behavior [23], as highlighted by electroantennography [24,25] and proboscis-extension reflex tests [25]. Protein families including odorant-binding proteins (OBPs) and chemosensory proteins (CSPs) [22] in the antennae were proven to deliver hydrophobic airborne molecules to olfactory receptors.

Moreover, the identification of the VOCs and the determination of the electroantennography (EAG) response may enhance our understanding of the chemical communication underlying *V. destructor*–host interactions within honey bee colonies and could offer valuable insights toward developing solutions that minimize our reliance on miticides [26].

With this aim, we conducted a three-tiered study asking the following questions: (i) Do the EAG responses of *A. cerana cerana* (*A. c. cerana*) and *A. mellifera ligustica* (*A. m. ligustica*) show differences in varroa VOCs? (ii) What compound(s) is/are the first chemical cue(s) for honey bees to recognize the parasitic mite’s presence? (iii) What genes are regulated in OBPs and CSPs? To answer these questions, the chemical ecology between the host honey bee and the parasite varroa mite was evaluated via EAG determination, gas chromatography–mass spectrometry (GC-MS) identification, and related gene expression profiles. This comparative study on varroa VOC recognition by the antennae of two major honey bee species may provide a better future understanding of the differences in their chemophysiology and behaviors.

## 2. Materials and Methods

### 2.1. Honey Bee Colonies and V. destructor Mites

Five colonies of each honey bee species were used in the experiment to elucidate differences between colonies. *A. c. cerana* and *A. m. ligustica* colonies were maintained at the apiary of Shandong Agricultural University from May to October 2023, when *V. destructor* was in its rapid reproductive phase. Adult workers were randomly collected from the combs. *V. destructor* mites were obtained from adult bees in five *A. m. ligustica* colonies. In detail, we collected the varroa-infested bees using tweezers and brushed the varroa mites into a collection box. This method avoids damaging the mites and prevents the introduction of other VOCs that could interfere with the results. It should be mentioned that varroa mites were absent from the *A. c. cerana* colonies, which exhibited greater resistance to varroa mite parasitism.

### 2.2. EAG Response to V. destructor VOCs

Fifteen bees of each species were collected and maintained in a wooden cage in a dark incubator (34 °C, 70% RH) and fed with 50% syrup *ad libitum*. These bees were used to test the EAG response differences between *A. m. ligustica* (*Aml*) and *A. c. cerana* (*Acc*). Additionally, thirty more bees of each species were used to test the EAG response of *A. m. ligustica* (*Aml*) and *A. c. cerana* (*Acc*) to three different concentrations (5, 50, and 500 μg/μL, *w*/*v*) of four individual VOCs selected from *V. destructor*. Up to 150 antennae were used in the EAG testing. The whole antennae were cut off and rapidly stabilized on two electrodes (Syntech, SpectraMax i3x, San Jose, CA, USA) using a conductive gel (Parker, Signa Gel, Fairfield, NJ, USA) and placed 1 cm away from the airflow outlet. The airflow controller (Syntech, DI-210) for the EAG experiment can convey the pulse airflow bypassing the mites (treatments) or blank (negative control) with a flow rate of 0.4 mL/s that lasted for 0.5 s, at intervals of 15 s applied after each pulse. One hundred and fifty *V. destructor* mites were randomly divided into fifteen groups with ten in each group, and kept at room temperature. In each trial, we transferred 10 mites to filter paper (0.8 cm × 1.0 cm) using sterilized tweezers. The EAG signal measurement system was placed in a rectangular box with a gate on a stable surface to reduce the vibration and airstream. We also measured the EAG responses to the top 4 VOCs of *V. destructor* with high content for which compound(s) could be sensitively recognized by the host. Individual volatile compounds were dissolved in liquid paraffin (Macklin, purity > 99%, Shanghai, China) to prepare three different concentrations (5, 50, and 500 μg/μL). Ten microliters of each solution were applied to the filter paper inside the pipettes connected to the airflow controller. Both air and liquid paraffin were used as controls. The EAG response to the different VOCs was measured in terms of the EAG amplitudes and depolarization and repolarization times [27] to characterize the response speed and recovery ability of the antennae to VOCs. By analyzing the differences in depolarization and repolarization times between *A. m. ligustica* and *A. c. cerana*, we aimed to investigate how these temporal parameters might correlate with their differing hygienic behaviors.

### 2.3. Identification of V. destructor VOCs

As one of the study aims was to obtain the VOCs, 900 *V. destructor* mites were collected and randomly divided into 3 groups with 300 mites in each group. The VOCs in the headspace were enriched and extracted by means of headspace solid-phase microextraction (HS-SPME, Supelco Inc., Bellefonte, PA, USA). The HS-SPME conditions were further optimized by carefully selecting the sample volume, temperature, and extraction time. During the experiment, the mites (300 individuals) were placed in a 1.5 mL vial (Agilent), and 50/30 μm DVB/CAR/PDMS fibers (Supelco Inc., Bellefonte, PA, USA) were then inserted into the vial for a 60 min extraction period at 65 °C. The samples were then removed and placed in the inlet of the tube column at 250 °C for 5 min desorption.

GC-MS (QP2010 Plus, Shimadzu Corporation, Kyoto, Japan) was performed for the qualitative and quantitative analysis of VOCs, with the following conditions: the GC capillary column was Rtx-5MS (Restek, Bellefonte, PA, USA); the inlet temperature was 250 °C; and helium was applied as a carrier gas (percentage purity ≥ 99.99%), with a constant flow rate of 30 cm/s and a purge flow of 2 mL/min. The linear velocity was set to 36.1 cm/s. The oven temperature program was set so that the temperature was at 40 °C for 5 min, then increased to 120 °C at 5 °C/min, then was maintained for 10 min, then increased to 250 °C at 15 °C/min, and then was maintained for 10 min. The column flow rate was 1 mL/min, the column head pressure was 49.7 kPa, the total flow rate was 8 mL/min, the split ratio was 5:1, and the scan range was 45–450 amu. The analysis of mass spectra was conducted utilizing NIST libraries, specifically, nist17, nist17s, and nist18.

### 2.4. VOCs Regulated the Gene Expression Profiles of Honey Bees

One hundred and fifty bees from both *A. c. cerana* and *A. m. ligustica* were maintained in wooden cages (20 cm × 15 cm × 10 cm) with transparent lids. These cages were then placed in an incubator (34 °C, 70% RH, dark) for 1 h with ethyl palmitate exposure (500 μg/μL in paraffin). Paraffin oil was used as a positive control to eliminate background effects. The antennae were quickly excised and frozen in liquid nitrogen, then stored at −80 °C until use. The total RNA was extracted using the TRnaZol RNA Kit (50T, New Cell & Molecular Biotech Co., Ltd., Suzhou, Jiangsu, China). cDNA was obtained via reverse transcription with the *Evo M-MLV* RT Master Mix Kit (Accurate Biology Co., Ltd., Changsha, Hunan, China). The primers of the selected genes, *AmelOBP*, *AmelCSP*, *AcerOBP*, and *AcerCSP*, were designed and synthesized by Shanghai Sangon Biotech (Songjiang, Shanghai, China) (Table 1), and Amel*β-actin* and Acer*β-actin* were used as the controls. Real-time PCR reactions were performed using QuantStudio 5 (ABI, Waltham, MA, USA) according to protocol.

### 2.5. Varroa Mites Regulate the Respiratory Metabolic Rate of Honey Bees

To investigate the effects of varroa mite parasitism on the respiratory metabolic rate of honey bees, we inoculated the bees with varroa mites before their cells sealed, continuing until sampling. Untreated healthy bees served as controls. Fifty bees at specific development stages (6-day old larvae, 4-day old pupae, new adults, and 6-day old adults) were collected from both *A. c. cerana* and *A. m. ligustica* and randomly divided into five replicates for the respiratory metabolic rate analysis using the insect respirometer system SMB-02 (Jinan, China). The parameters were as follows: an air flow of 500 mL/min with a time interval of 10 s; a respiratory chamber volume of 0.1 L; a temperature of 35 ± 1 °C; and humidity at 40%. After preheating the instrument for 3 min and stabilizing the CO_2_ flow, 10 bees were placed into the chamber to test the change in CO_2_ concentration within 10 s. The calculation formula is as follows:

R (mg/g·h) = [V · M · (w_2_ − w_1_)]/(V_0_ · m · t)

where R indicates the respiratory metabolic rate (mg/g·h); V indicates the volume of the respiratory chamber (L); M indicates the molar mass of CO_2_ (g/mol); w_2_ and w_1_ refer to the concentration of CO_2_ in the respiratory chamber at the end or before of the measurement (%); V_0_ is the molar volume of carbon dioxide at the measured temperature (L/mol); m is the weight of the measured sample (g); and t is the determination time of the sample (h).

### 2.6. Statistical Analysis

The results are presented as means ± SEMs, with three independent replicates in each experiment. The statistical analysis tool SPSS 27.0 (Chicago, IL, USA) was used, and significant differences were identified by means of Tukey’s test and an independent samples *t*-test for EAG and gene expression analysis and by means of one-way ANOVA for the comparative analysis of the respiratory metabolic rate between the two species, considering a *p*-value < 0.05 as being statistically significant; such differences are marked with letters and asterisks. The same and different letters indicate non-significant and significant differences, respectively. GraphPad PRISM 9.5 (San Diego, CA, USA) was also used for data analysis and figure layout. Virtual screenings were conducted using AutoDock Vina 1.2.0 software (Center for Computational Structural Biology, La Jolla, CA, USA) combined with vina (https://vina.scripps.edu/downloads/, accessed on 23 October 2024), MGLtools (https://ccsb.scripps.edu/mgltools/downloads/, accessed on 23 October 2024) and PyMOL 2.5.0 (Schrodinger LLC, San Diego, CA, USA).

## 3. Results

### 3.1. EAG Response Differences Between A. c. cerana and A. m. ligustica

**Figure 1:**
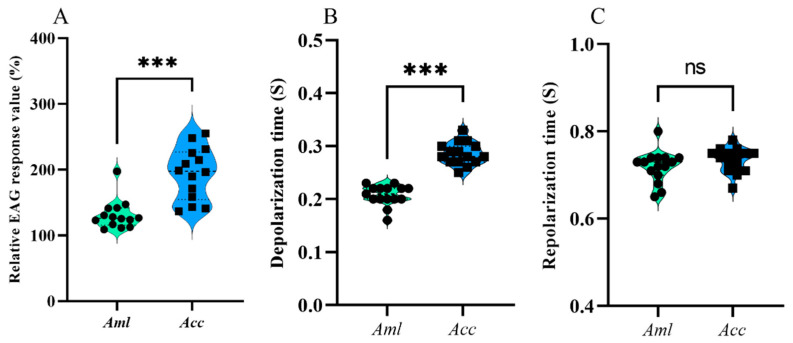
The EAG response characteristics of *A. m. ligustica* (*Aml*) and *A. c. cerana* (*Acc*) to the VOCs of *V. destructor*. (**A**) Relative EAG response value, (**B**) depolarization times, and (**C**) repolarization times between *Aml* and *Acc*. *** Indicates *p* < 0.001; ns indicates no statistical difference.

The EAG responses of *A. c. cerana* were significantly higher than those of *A. m. ligustica* (*p* < 0.001) (Table S1), indicating that *A. c. cerana* may have more acute sensitivity to the VOCs from *V. destructor* (Figure 1A). The depolarization of *A. c. cerana* was significantly longer than that of *A. m. ligustica* (*p* < 0.001), but there was no significant difference in repolarization (Figure 1B,C). The results suggest that the antennae of the two species were responsive to *V. destructor* VOCs, but *A. c. cerana* was more sensitive. Because the VOCs are mixtures, it is not clear which compound(s) is/are recognized by the bees’ antennae.

### 3.2. Identification of V. destructor VOCs

The VOCs released by the mite *V. destructor* comprised 15 compounds, including ethyl palmitate (accounting for 29.72%), isoamyl alcohol (14.18%), nonanal (13.52%), and ethyl oleate (7.63%) (Table 2).

### 3.3. EAG Responses of Honey Bees to V. destructor VOCs

Different compounds varied strongly in their effects on the EAG responses of *A. c. cerana* and *A. m. ligustica*. The most responsive signals were observed for ethyl palmitate in the VOCs, and the responses increased with the concentration. The EAG responses of *A. c. cerana* to different concentrations of the VOCs from *V. destructor* were generally stronger than those of *A. m. ligustica*.

**Figure 2:**
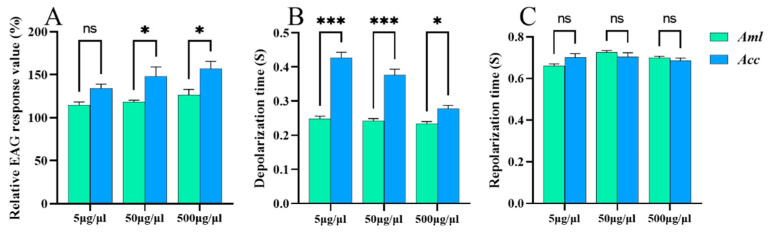
The EAG response of *A. m. ligustica* (*Aml*) and *A. c. cerana* (*Acc*) to three different concentrations of ethyl palmitate. (**A**) The relative EAG response value of bees to ethyl palmitate. (**B**) The depolarization time. (**C**) The repolarization time. * Indicates *p* < 0.05; *** indicates *p* < 0.001; ns indicates no statistical difference.

The EAG of ethyl palmitate was significantly higher in *A. c. cerana* than in *A. m. ligustica* at all three concentrations (5, 50, and 500 μg/μL) (*p* < 0.05) (Figure 2A), and the depolarization time of the antennal potential was significantly longer in *A. c. cerana* than in *A. m. ligustica* when stimulated by ethyl palmitate (*p* < 0.001) (Figure 2B). As shown in Figure 2C, there was no significant difference in the repolarization times of the antennal potential between the two species (*p* > 0.05).

**Figure 3:**
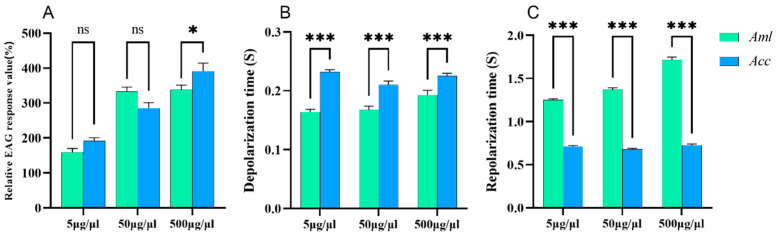
The EAG response of *A. m. ligustica* and *A. c. cerana* to three different concentrations of isoamyl alcohol. (**A**) The relative EAG response values of bees to isoamyl alcohol. (**B**) The depolarization times. (**C**) The repolarization times. * Indicates *p* < 0.05; *** indicates *p* < 0.001; ns indicates no statistical difference.

The antennal potential response value of isoamyl alcohol in *A. c. cerana* was significantly higher than that in *A. m. ligustica* bees at 500 μg/μL (*p* < 0.05); however, no significant results were found at 5 and 50 μg/μL (*p* > 0.05) (Figure 3A). The depolarization time of the antennal potential of *A. c. cerana* after stimulation with isoamyl alcohol was significantly longer than that of *A. m. ligustica* (*p* < 0.05) (Figure 3B), and the repolarization time of the antennal potential stimulated by isoamyl alcohol was significantly longer than that of *A. c. cerana* (*p* < 0.001) (Figure 3C).

**Figure 4:**
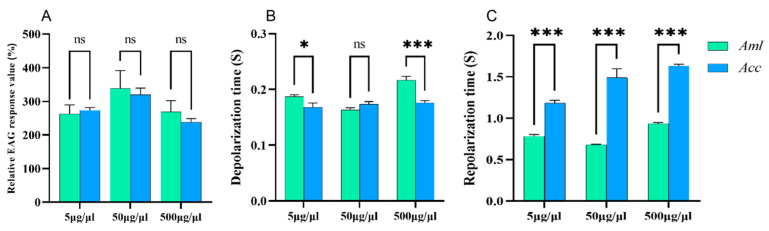
The EAG response of *A. m. ligustica* (*Aml*) and *A. c. cerana* (*Acc*) to three different concentrations of nonanal. (**A**) The relative EAG response values of bees to nonanal. (**B**) The depolarization times. (**C**) The repolarization times. * Indicates *p* < 0.05; *** indicates *p* < 0.001; ns indicates no statistical difference.

Nonanal exposure did not cause a significant difference in EAG response at the three concentrations (Figure 4A). However, differences in depolarization times emerged between *A. m. ligustica* and *A. c. cerana* at concentrations of 5 μg/μL (*p* < 0.05) and 500 μg/μL (*p* < 0.001) (Figure 4B). Furthermore, the repolarization time of *A. c. cerana* was significantly longer than that of *A. m. ligustica* (*p* < 0.001) (Figure 4C).

**Figure 5:**
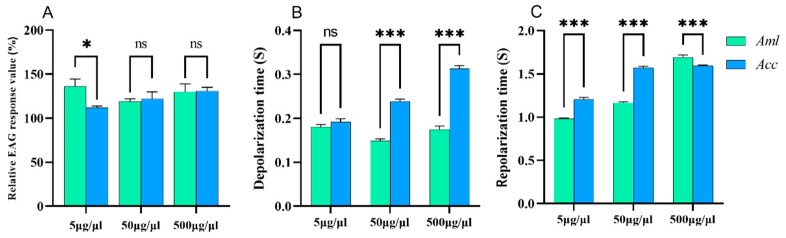
The EAG response of *A. m. ligustica* (*Aml*) and *A. c. cerana* (*Acc*) to three different concentrations of ethyl oleate. (**A**) The relative EAG response values of bees to ethyl oleate. (**B**) The depolarization times. (**C**) The repolarization times. * Indicates *p* < 0.05; *** indicates *p* < 0.001; ns indicates no statistical difference.

Ethyl oleate treatments led to a significantly higher EAG response in *A. m. ligustica* than in *A. c. cerana* at a concentration of 5 μg/μL (*p* < 0.05), but no significant results were found at concentrations of 50 and 500 μg/μL (*p* > 0.05) (Figure 5A). The depolarization times of the antennal potential in *A. c. cerana* was significantly longer than that in *A. m. ligustica* at concentrations of 50 and 500 μg/μL (*p* < 0.05), except for 5 μg/μL (*p* > 0.05) (Figure 5B). The repolarization times of the antennal potential in *A. c. cerana* was significantly longer than that in *A. m. ligustica* at concentrations of 5 and 50 μg/μL (*p* < 0.001), as well as at a concentration of 500 μg/μL (*p* < 0.01) (Figure 5C).

### 3.4. VOCs Regulate Host Gene Expression Profiles

Through virtual screening, the proteins AcerCSP1 and AcerOBP21 in *A. c. cerana* and AmelCSP1 and AmelOBP8 in *A. m. ligustica* were found to have a stronger affinity for ethyl palmitate (affinity < −7 kcal/mol) (Table 3).

**Figure 6:**
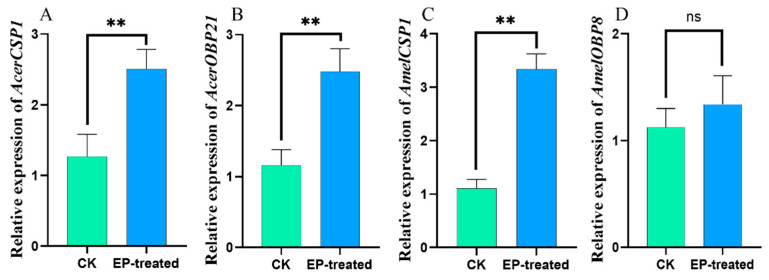
The effect of exposure to ethyl palmitate on the expression levels of (**A**) *AcerCSP1*, (**B**) *AcerOBP21*, (**C**) *AmelCSP1*, and (**D**) *AmelOBP8*. ** Indicates *p* < 0.01; ns indicates no statistical difference.

Ethyl palmitate exposure significantly enhances the gene expressions of *AcerCSP1*, *AcerOBP21* (*p* < 0.01), and *AmelCSP1* (*p* < 0.001), but not *AmelOBP8* (*p* = 0.5) (Figure 6).

### 3.5. Varroa Mites Regulate the Respiratory Metabolic Rate

**Figure 7:**
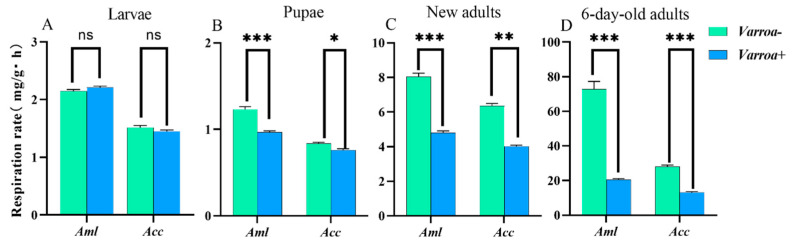
The respiratory metabolic intensity of bees at different ages. (**A**) The respiratory metabolic intensity of 6-day-old larvae. (**B**) The respiratory metabolic intensity of 4-day-old pupae. (**C**) The respiratory metabolic intensity of a 1-day-old worker. (**D**) The respiratory metabolic intensity of a 6-day-old worker. * Indicates *p* < 0.05; ** indicates *p* < 0.01; *** indicates *p* < 0.001; ns indicates no statistical difference.

**Figure 8:**
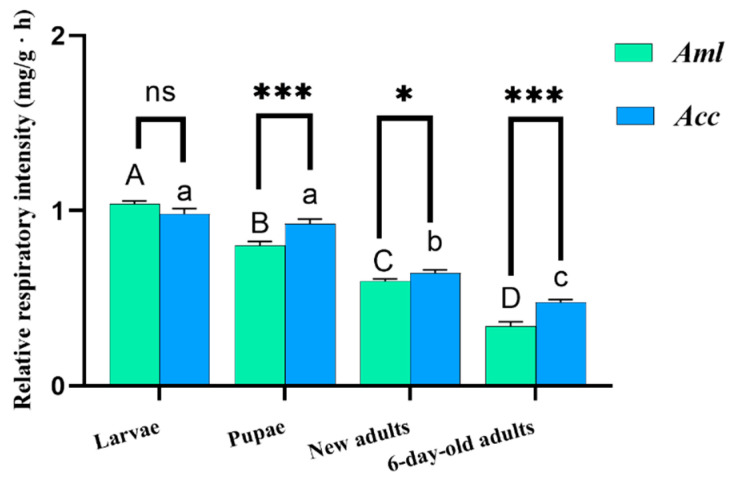
Relative respiratory metabolic intensity of bees at different ages. Identical letters indicate no significant difference (*p* > 0.05), and different letters represent significant differences (*p* < 0.05). To clarify, capital letters (A, B, C, and D) used for comparisons of different development stages in *Aml*, while lowercase letters (a, b, and c) used for those in *Acc*. * Indicates *p* < 0.05; *** indicates *p* < 0.001; ns indicates no statistical difference.

Under parasitic stress from varroa mites, there was no significant change in the respiratory metabolic rate in larvae (*p* > 0.05) (Figure 7A), but the rate decreased significantly in pupae (Figure 7B), as well as in new adults (Figure 7C) and 6-day-old adult bees (*p* < 0.001) (Figure 7D). This indicates that varroa influenced the respiratory metabolic capacity of the bees and caused further damage with time. The respiratory metabolic rate of the *A. mellifera* bee decreased more than that of the *A. cerana* bee during particular developmental stages (Figure 8).

## 4. Discussion

*V. destructor* injures host bees by ingesting fat bodies; therefore, these mites are blamed for increased annual winter colony mortalities [28], as well as reduced bee health and overwinter survival [29]. Interestingly, the honey bee *A. c. cerana*, identified as the initial host of the ectoparasitic mite *V. destructor* [30] approximately 70 years ago, exhibits reduced vulnerability to the mite due to their coexistence over an extended evolutionary period [31]. Shortly after transferring its host to the Western honey bee [32], *V. destructor* rapidly disseminated across the globe [31]. Honey bee colonies typically face a substantial threat once they become parasitized by *V. destructor* due to its rapid reproduction rate, especially in the absence of effective control measures [28,33]. Therefore, it is crucial that we implement strategies to manage and mitigate the impact of *V. destructor* on affected colonies [33]. This study could be used to fill some current gaps in knowledge about the host–parasite interactions.

The diet of *V. destructor* varies according to the stage of its host’s life cycle. *V. destructor* mites primarily consume the fat body tissue of adult honey bees [29] rather than the hemolymph of honey bees’ pupae during their reproductive stage [34]. Therefore, it is crucial for *V. destructor* that it can detect and interpret the chemical cues of the host to complete its life cycle [12,35].

Chemical communication is widely deployed across bee castes and individuals, as well as hygienic behavior, known as VSH behavior. VSH encompasses several stages, beginning with the identification of a compromised brood, followed by the selective uncapping of cells infested with varroa mites, and culminating in the targeted sacrifice of specific brood. Each of these stages may be initiated by different compounds. For instance, the initial stage may rely on the detection of highly volatile substances like ocimene, which assist bees in pinpointing areas with damaged brood [36]. Subsequently, the identification of less volatile compounds, including alkenes or oleic acid, enables the precise targeting of the most severely affected brood [37]. Moreover, bees can detect varroa mite-infested cells using two unsaturated hydrocarbons, (*Z*)-6-pentadecene and (*Z*)-10-tritriacontene, which are emitted by the brood themselves [38]. Nevertheless, the most complex aspect of this process is arguably the selective sacrifice of the infested brood. So far, several compounds originating from varroa-parasitized brood cells were described as triggering hygienic behaviors in workers [15]. The VOCs of *V. destructor* are unexplored, in contrast to the VOCs of adult bees and their broods.

*V. destructor* VOCs are important semiochemical cues in eliciting behavioral responses in honey bees for initial recognition and then hygiene behaviors. Female varroa mites attach to adult honey bees, facilitating their dispersal to new hosts. Although bee broods produce a higher number of VOCs that allow information to spread rapidly over a long distance within the colony, there are currently few accounts of *V. destructor* VOC-mediated interactions between honey bees and varroa available. Testing the locomotion behavioral responses of *V. destructor* to these VOCs in a concentration-dependent manner may help to identify which chemicals should be the focus of future investigations into managing *V. destructor* [26]. Previous studies have shown that the sensitive hygienic behavior of bees toward varroa mites can be triggered by changes in brood pheromones related to diseased larvae or pupae [39] or by chemical cues such as VOCs emitted after varroa parasitism [37]. Substances released during the egg-laying behavior of varroa mites can also trigger sensitive hygienic behavior in bees [40]. However, varroa mites were observed to imitate the cuticular hydrocarbons (CHCs) of their host honey bees, which allows them to evade the hygienic behavior exhibited by their hosts [41].

Martin et al. reported that varroa VOC profiles vary depending on the developmental stage of the host, as detected via GC-MS methods [42]. So far, studies on VOCs in *V. destructor* have mainly focused on the developmental and reproductive stages, and little is known about semiochemical application prospects, whereas sensitivity to the VOCs of *V. destructor* in honey bees has been the subject of fewer research.

In this study, we utilized HS-SPME and GC-MS to characterize the headspace volatiles of several varroa mite samples. A total of fifteen VOCs from seven specific chemical families were identified, with fatty acid esters accounting for the highest percentage at 48.79%. Fatty acids and their respective, i.e., methyl and ethyl, esters were shown to play a vital role in bee communication. Related studies have isolated ten fatty acid esters as bee larval pheromones, including methyl palmitate, ethyl palmitate, methyl oleate, ethyl oleate, methyl stearate, ethyl stearate, methyl linoleate, ethyl linoleate, methyl linolenate, and ethyl linolenate [43], which are closely related to worker bee capping and nursing behaviors and are secreted by the salivary glands of bee larvae [44]. Fatty acid methyl esters and ethyl esters peak when bee larval cells are capped and quickly decrease after capping is completed [45]. Varroa mites choose suitable parasitic hosts based on the fatty acid esters released by bees [46]. Additionally, fatty acids and fatty acid esters play a significant role in the reproduction of female varroa mites. Using SPME-GC-MS, a study identified 2-hydroxyacetic acid in the headspace volatiles of royal jelly, which can induce varroa reproduction [47]. Another study found that octanoic acid in royal jelly acts as a repellent for varroa mites [48]. Pentane-extracted polar components from bee larval cuticles can trigger egg-laying behavior in female varroa mites [49]. Additional studies have demonstrated that palmitic acid, ethyl palmitate, and ethyl stearate serve as components of female varroa sex pheromones, facilitating their egg-laying behavior [50]. Moreover, ethyl palmitate was found in the volatiles of various insects, including in the pheromone-secreting glands of navel orange worm larvae (*Amyelois transitella*) [51] and in the eggs of the cabbage moth (*Mamestra brassicae*) [52].

Moreover, numerous organisms employ chemical mimicry by replicating the odors of other creatures or objects to fulfill certain objectives. For instance, the beetle *Metoecus paradoxus* mimics the pheromones of the *Vespula vulgaris* wasp queen by consuming its larvae thereby evading wasp attacks [53]. Certain ant-dispersed plants also employ chemical imitation to mimic ant nest cues, leading ants to spread their seeds without receiving any reward [54]. In the dark environment of a beehive, chemical communication predominates. Varroa mites also use chemical mimicry to evade detection by worker bees, resulting in their chemical cues differing from those of their parasitic hosts. Research indicates that varroa mites modify their CHC profiles to match those of their host’s CHCs [55]. This chemical mimicry allows the varroa mites to blend in with their host’s chemical signature, potentially helping them avoid detection and removal by the bees. Varroa mites also rapidly adapt their chemical mimicry when transitioning between different life stages or groups [41]. This adaptability is key to their survival and reproductive success as it allows them to remain undetected by the host bees throughout their life cycle. Moreover, varroa mites from different bee species (*A. m. ligustica* and *A. c. cerana*) may have even greater differences in both CHCs and VOCs. However, obtaining the varroa mite parasitizing *A. c. cerana* is challenging. Considering this, we mixed the varroa mites collected from the different colonies of *A. m. ligustica* to minimize variation between individuals and performed multiple replicates. EAG is frequently used in the screening of insect VOCs, often in combination with GC-MS for simultaneous identification and screening. For instance, OcomOBP7 in a beetle (*Ophraella communa*) binds broadly to the volatiles of common ragweed (*Ambrosia artemisiifolia*). EAG verification following RNAi showed a significant reduction in response to α-pinene and myrcene, indicating the localization of the ragweed protein [56]. EAG was used to screen for active substances in the volatile chemicals of bed bug nests to create lure traps [57]. In our study, the electrophysiological response of *A. c. cerana* to the VOCs of varroa mites was significantly higher than that of *A. m. ligustica*, indicating that the VOCs emitted by varroa mites could cause a stronger olfactory signal change in *A. c. cerana*. Research has observed that the olfactory signal changes associated with varroa mite infestation from the chemical basis for triggering bee hygienic behavior [58]; there is a close relationship between hygienic behavior and olfactory sensitivity [59]. Therefore, we speculate that the stronger olfactory signal change in *A. c. cerana* in response to varroa mite VOCs in experiments might be the key to its stronger hygienic behavior.

In this experiment, we selected four substances with relatively high content, including ethyl palmitate, isoamyl alcohol, nonanal, and ethyl oleate for EAG measurements at different doses. When bee antennae were stimulated with varying doses of ethyl palmitate, *A. cerana* showed significantly higher EAG response values compared to *A. mellifera* and, likewise, higher performance when directly stimulated by varroa mite odors. However, the two bee species had different trends in the duration of depolarization. Wang et al. also reported that the most prevalent and volatile of the substances examined, isopentyl acetate (IPA), is present in all three species of honey bee and was avoided by *A. cerana* foragers. Interestingly, gamma-octanoic lactone (GOL), an odor component that is more than 150 times less volatile than IPA, was likewise avoided by *A. cerana*. *A. cerana* antennae are ten times more sensitive to GOL than to other chemicals examined, according to EAG [60]. In the present study, *A. c. cerana* showed a significantly longer depolarization time compared to *A. m. ligustica*, while there was no significant difference in the repolarization time between the species. The differential response to these esters between *A. c. cerana* and *A. m. ligustica* could explain the more proactive hygienic behavior in *A. c. cerana*, to a certain extent. Therefore, *A. mellifera* exhibits high mite levels and high colony death rates because it has not yet developed a symbiotic parasite–host relationship [61].

OBPs and CSPs exhibit selective binding characteristics to odor molecules. For example, AeOBP22 in a mosquito (*Aedes aegypti*) undergoes specific conformational changes at the protein terminus to create a higher affinity binding cavity [62]. Research indicates that exposure to specific odors significantly upregulates or downregulates the expression of *OBPs* and *CSPs* involved in the recognition process. When cotton boll weevils (*Anthonomus grandis*) were exposed to informative compounds, *OBPs* in the female antennae showed various levels of upregulation and downregulation, whereas males only exhibited downregulation in response to certain compounds, which is consistent with their chemical ecological behavior [63]. Four-day-old larvae of *Spodoptera litura* exposed to sex pheromones showed the significant upregulation of seven OBPs, which displayed strong binding characteristics. These OBPs are actively involved in the perception of sex pheromones [64]. In this study, exposure to ethyl palmitate resulted in the significant upregulation of *AcerCSP1*, *AcerOBP21*, and *AmelCSP1*, while *AmelOBP8* showed no significant changes in expression. Thus, AcerCSP1, AcerOBP21, and AmelCSP1 might be involved in the recognition response to ethyl palmitate in *A. c. cerana* and *A. m. ligustica*.

## 5. Conclusions

Our study was centered around characterizing the VOCs of the adult *V. destructor* to serve as a foundation for future research. In general, our findings suggest that the VOCs could induce chemoreception signal transduction by altering the expression profiles of OBPs and CSPs and consequently evoking chemosensory abilities in honey bees, providing new insights into the prevention and control of *V. destructor* in beekeeping. This study offers a better understanding of the chemical communication between honey bees and their epizoic mite. To our knowledge, this is the first attempt to explore the relationship between *V. destructor* VOCs and honey bees. Improving our understanding of the chemical ecology interactions between *V. destructor* and its host may offer new perspectives toward reducing the use of miticides.

## Figures and Tables

**Table 1:** Information for the primers used in this study.

Gene Names	GenBank Accession Number	Primer Sequence (5′–3′)
*AmelCSP1*	NM_001077820	F: CGTGGACATATGCTGAGGAACTT R: TCTGCTAATTTGTCCAAGTTTTGT
*AmelOBP8*	NM_001171044	F: TTGCAGCAAGAAGAACGACACC R: CAACCTCGCATCCCTCCGTAG
*AcerCSP1*	FJ157352	F: ACGTGGACATATGCTGAGGAA R: GGAGTTAAACAAGGGCCTGC
*AcerOBP21*	KP717063	F: TGCGTTGGTGCATTGACACT R: AGACGAATGTTGGCATCAGTGA
*Amelβ-actin*	NM_001185145	F: CCGTGATTTGACTGACTACCT R: AGTTGCCATTTCCTGTTC
*Acerβ-actin*	HM640276.1	F: TTATATGCCAACACTGTCCTTT R: AGAATTGATCCACCAATCCA

**Table 2:** Acquisition of VOCs in headspace of *V. destructor*.

#	Molecular Formula	VOC Name	CAS #	Retention Time (min)	Relative Standard Deviation (%)	Relative Content (%)	Retention Index
1	C_6_H_14_O_2_	Acetaldehyde diethyl acetal	105-57-7	3.961	0.710	4.50 ± 0.90	705
2	C_5_H_12_O	Isoamyl alcohol	123-51-3	4.052	0.711	14.18 ± 5.13	697
3	C_7_H_14_O_2_	Ethyl 2-methyl butyrate	7452-79-1	8.967	0.242	1.03 ± 0.16	820
4	C_10_H_16_	DL-Limonene	138-86-3	17.056	0.030	1.98 ± 1.36	1018
5	C_9_H_18_O	Nonanal	124-19-6	19.849	0.012	13.52 ± 2.79	1104
6	C_10_H_20_O_2_	Ethyl octanoate	106-32-1	22.862	0.003	1.99 ± 0.10	1183
7	C_10_H_12_O	Estragole	140-67-0	22.937	0.026	6.87 ± 2.13	1172
8	C_15_H_32_	2,6,11-Trimethyldodecane	3891-98-3	25.345	0.014	2.77 ± 0.96	1320
9	C_14_H_28_O_2_	Ethyl laurate	106-33-2	38.306	0.030	2.76 ± 0.22	1580
10	C_15_H_26_O	α-Cedrol	77-53-2	38.430	0.005	5.83 ± 1.29	1543
11	C_15_H_26_O	β-Cineole	470-82-6	39.150	0.000	1.55 ± 0.10	1593
12	C_16_H_32_O_2_	Ethyl myristate	124-06-1	40.627	0.030	4.49 ± 0.71	1779
13	C_18_H_34_O_2_	Ethyl 9-hexadecenoate	54546-22-4	42.087	0.021	1.17 ± 0.37	1986
14	C_18_H_36_O_2_	Ethyl palmitate	628-97-7	42.210	0.015	29.72 ± 9.89	1978
15	C_20_H_38_O_2_	Ethyl oleate	111-62-6	43.507	0.013	7.63 ± 1.25	2185

**Table 3:** Docking fraction of olfactory-related proteins in antennae with ethyl palmitate.

Protein Name	Affinity (kcal/mol)	Protein Name	Affinity (kcal/mol)
AmelCSP 1	−7.8	AcerCSP 1	−8.1
AmelCSP 3	−6.2	AcerCSP 3	−6.5
AmelOBP 1	−5.8	AcerOBP 1	−5.8
AmelOBP 2	−4.7	AcerOBP 2	−4.7
AmelOBP 4	−5.2	AcerOBP 4	−6.1
AmelOBP 6	−6.7	AcerOBP 5	−5.2
AmelOBP 8	−7.2	AcerOBP 14	−4.0
AmelOBP 11	−5.7	AcerOBP 15	−5.8
AmelOBP 16	−6.0	AcerOBP 19	−4.2
AmelOBP 19	−4.8	AcerOBP 21	−7.4
AmelOBP 21	−4.1		

Note: The smaller the affinity, the stronger the binding force. The following is a rule of thumb: affinity > −4 kcal/mol indicates an extremely weak binding force or is considered as no binding; −7 kcal/mol < affinity ≤ −4 kcal/mol indicates a moderate binding force; and affinity ≤ −7 kcal/mol indicates a strong adhesion.

## Data Availability

The original contributions presented in the study are included in the article; further inquiries can be directed to the corresponding authors.

## Supplementary Materials

The following supporting information can be downloaded at: https://www.mdpi.com/article/10.3390/biom15010066/s1, Table S1: The EAG response data.
